# Emerging peptide and neuroendocrine strategies for TRT-induced reproductive suppression and functional male hypogonadism: a narrative review

**DOI:** 10.3389/frph.2026.1914709

**Published:** 2026-08-14

**Authors:** Ana-Maria Cretu, Ahmed Adel Mansour Kamar, Alin Ciobica, Dragos Puia, Mara Doroftei, Bogdan Doroftei

**Affiliations:** 1 Origyn Fertility Center, Iași, România; 2 Faculty of Medicine, Grigore T. Popa University of Medicine and Pharmacy Iasi, Iași, Romania; 3 Department Biology, Faculty of Biology, Doctoral School, Alexandru Ioan Cuza University, Iași, Romania; 4 Department Biology, Faculty of Biology, Alexandru Ioan Cuza University (UAIC), Iași, Romania; 5 Department of Urology, “Dr. C.I. Parhon” Clinical Hospital, Iași, Romania; 6 Spitalul Clinic de Obstetrică și Ginecologie “Cuza Vodă”, Iași, Romania

**Keywords:** fertility restoration, hypothalamic-pituitary-testicular axis, male infertility, peptide therapeutics, reproductive endocrinology, spermatogenesis, testicular atrophy, testosterone replacement therapy

## Abstract

The growing burden of male hypogonadism has raised concerns regarding the potential impact of modern lifestyles and environmental exposures on male reproductive health. Testosterone replacement therapy (TRT) is widely used for the treatment of male hypogonadism. However, prolonged TRT may suppress the hypothalamic-pituitary-testicular (HPT) axis, resulting in reduced gonadotropin production, decreased intratesticular testosterone levels, impaired spermatogenesis, testicular atrophy, and compromised fertility. Current management strategies, including human chorionic gonadotropin (hCG), follicle-stimulating hormone (FSH), and selective estrogen receptor modulators (SERMs), can support recovery in some patients, but their effectiveness remains variable. This narrative review examines the emerging role of peptide and neuroendocrine strategies in TRT-induced reproductive suppression and functional male hypogonadism. A structured literature search was conducted to identify experimental, translational, and clinical studies investigating reproductive peptides and related neuroendocrine pathways. Particular attention was given to kisspeptin-based therapies, gonadotropin-releasing hormone (GnRH)-related approaches, neurokinin signaling pathways, and other developing interventions with potential relevance to male reproductive health. Current evidence suggests that peptide-based therapies may support endogenous hormonal signaling through targeted activation of the HPT axis. However, direct evidence demonstrating restoration of spermatogenesis or fertility following TRT-induced suppression remains limited, and important questions regarding long-term efficacy, safety, treatment protocols, and patient selection remain unresolved. Emerging peptide therapeutics represent a growing area of interest in reproductive endocrinology and may expand future treatment options for selected patients. Nevertheless, further well-designed clinical studies are required to establish their long-term efficacy, safety, and role in restoring reproductive function following TRT-induced suppression.

## 1 Introduction

Testosterone deficiency comprises a heterogeneous group of clinical conditions characterized by deficient testosterone production and associated clinical manifestations, including reduced libido, erectile dysfunction, decreased muscle mass, impaired bone health, metabolic disturbances, fatigue, and reduced quality of life (1–5). The prevalence of male reproductive disorders has increased in recent decades, with growing evidence suggesting contributions from aging, obesity, metabolic dysfunction, environmental endocrine disruptors, and other lifestyle-related factors affecting reproductive health (6–8).

Testosterone replacement therapy (TRT) remains the cornerstone of treatment for appropriately selected men with testosterone deficiency and provides substantial clinical benefits when prescribed according to current guideline recommendations (1, 2, 4, 5). However, exogenous testosterone suppresses the hypothalamic–pituitary–testicular (HPT) axis through negative feedback mechanisms, resulting in reduced gonadotropin-releasing hormone (GnRH), luteinizing hormone (LH), and follicle-stimulating hormone (FSH) secretion (9–11). Consequently, intratesticular testosterone concentrations decline, leading to impaired spermatogenesis, testicular atrophy, and compromised fertility despite normalization of serum testosterone levels (9–18, 39).

This limitation has become increasingly important as TRT use expands among younger men and those wishing to preserve future fertility. Available recovery strategies, including human chorionic gonadotropin (hCG), gonadotropins, selective estrogen receptor modulators (SERMs), and aromatase inhibitors, may support restoration of endogenous reproductive function (10, 11, 13–21); however, treatment responses remain variable, and spontaneous recovery after TRT discontinuation may also occur with highly variable timing (9, 12). Fertility preservation should therefore be considered before initiating TRT in men desiring future fertility, in accordance with current clinical guidelines (21, 22).

Recent advances in reproductive neuroendocrinology have identified several peptide-based pathways that regulate reproductive function at the level of the hypothalamus and pituitary gland (23–27). Among these, kisspeptin has emerged as a master regulator of GnRH secretion and reproductive axis activation (23–26, 28, 29). Human studies have demonstrated that kisspeptin administration stimulates LH and testosterone secretion, increases LH pulse frequency, and preserves physiological reproductive signaling (28, 30–35). Additional peptide-based approaches involving GnRH, neurokinin B, and KNDy neuron signaling pathways have further expanded interest in treatment strategies aimed at restoring endogenous reproductive function rather than replacing hormones directly (23–27).

These developments have introduced a promising therapeutic concept in male reproductive medicine by exploring strategies to enhance endogenous HPT-axis function beyond conventional hormone replacement. Accordingly, peptide therapeutics may support recovery of endogenous HPT-axis function following TRT-induced reproductive suppression and provide fertility-preserving therapeutic alternatives for selected men with functional male hypogonadism. Therefore, this narrative review evaluates the biological rationale, current evidence, therapeutic potential, and limitations of emerging peptide and neuroendocrine strategies in TRT-induced reproductive suppression and functional male hypogonadism, with particular emphasis on kisspeptin-based therapies, GnRH-related approaches, neurokinin signaling pathways, and fertility preservation.

**Figure 1:**
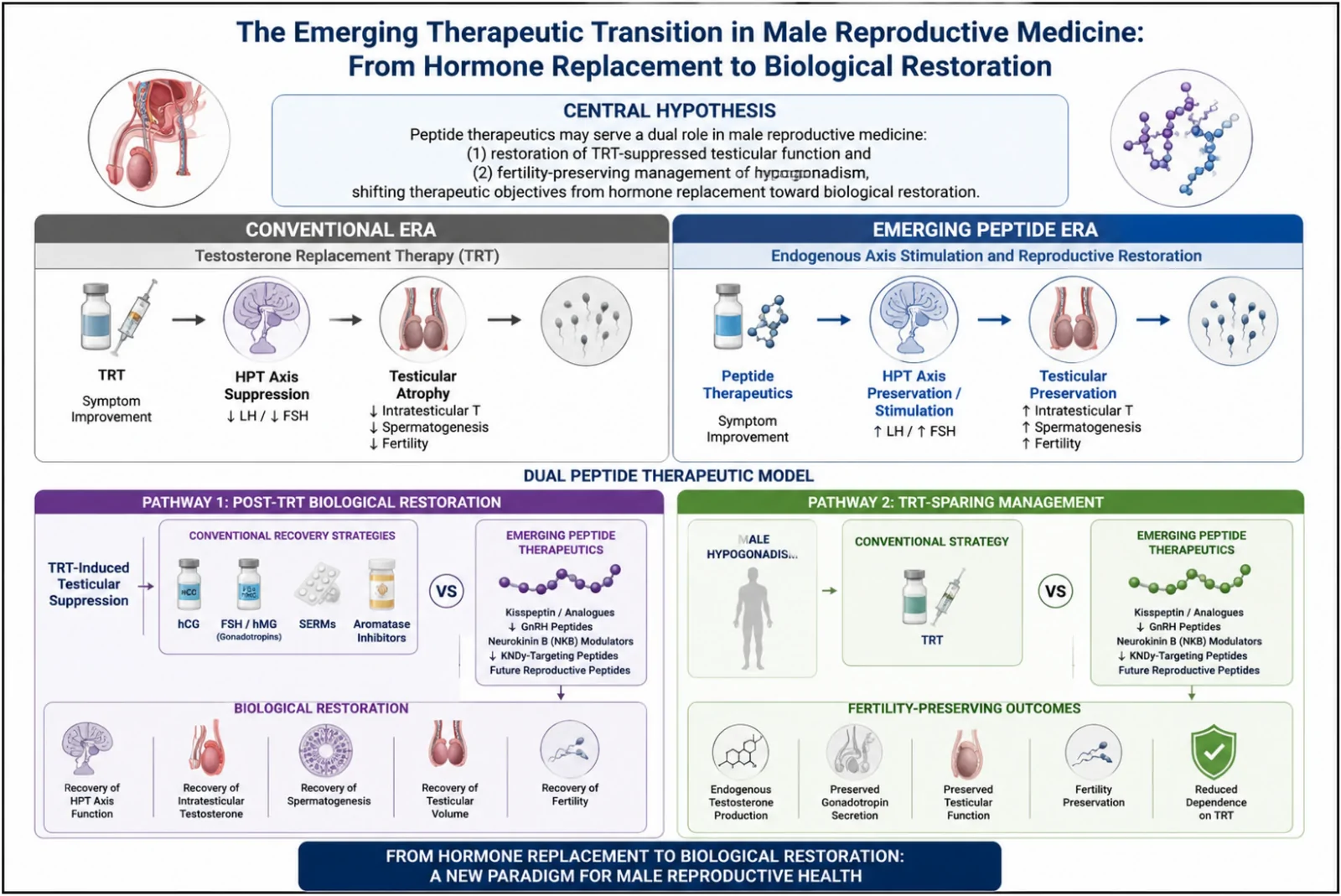
Conceptual framework of emerging peptide-based strategies in male reproductive medicine: From hormone replacement to biological restoration.

The conceptual framework proposed in this review is illustrated in Figure 1, highlighting emerging peptide-based strategies that may support endogenous HPT-axis function alongside current therapeutic approaches.

### 1.1 Research questions

#### 1.1.1 Primary research question

What is the potential role of peptide therapeutics as adjunctive or future alternatives for the restoration of TRT-suppressed testicular function?

#### 1.1.2 Secondary research questions

What biological mechanisms support the use of peptide therapeutics for restoration of hypothalamic–pituitary–testicular (HPT) axis function?
Can peptide-based therapies provide fertility-preserving alternatives or adjuncts to conventional testosterone replacement therapy in selected men with functional male hypogonadism?

## 2 Methodology and literature search strategy

This study was conducted as a narrative, non-systematic review. As the objective was to provide a comprehensive and clinically focused overview of emerging peptide-based therapeutic approaches in male reproductive medicine, protocol registration and formal risk-of-bias assessment were not performed.

A structured literature search was undertaken using PubMed/MEDLINE, Scopus, Web of Science, Embase, and Google Scholar. Search terms and Medical Subject Headings (MeSH) included combinations of: testosterone replacement therapy, hypogonadism, testicular atrophy, testicular suppression, spermatogenesis, male infertility, fertility restoration, peptide therapeutics, kisspeptin, gonadotropin-releasing hormone (GnRH), gonadorelin, neurokinin B, reproductive endocrinology, and the hypothalamic–pituitary–testicular (HPT) axis. The literature search was last updated in July 2026.

The search focused on publications in English and included original clinical studies, translational investigations, mechanistic studies, clinical guidelines, and high-quality review articles relevant to male reproductive endocrinology. Articles were screened for relevance based on title, abstract, and full-text review when appropriate.

**Figure 2:**
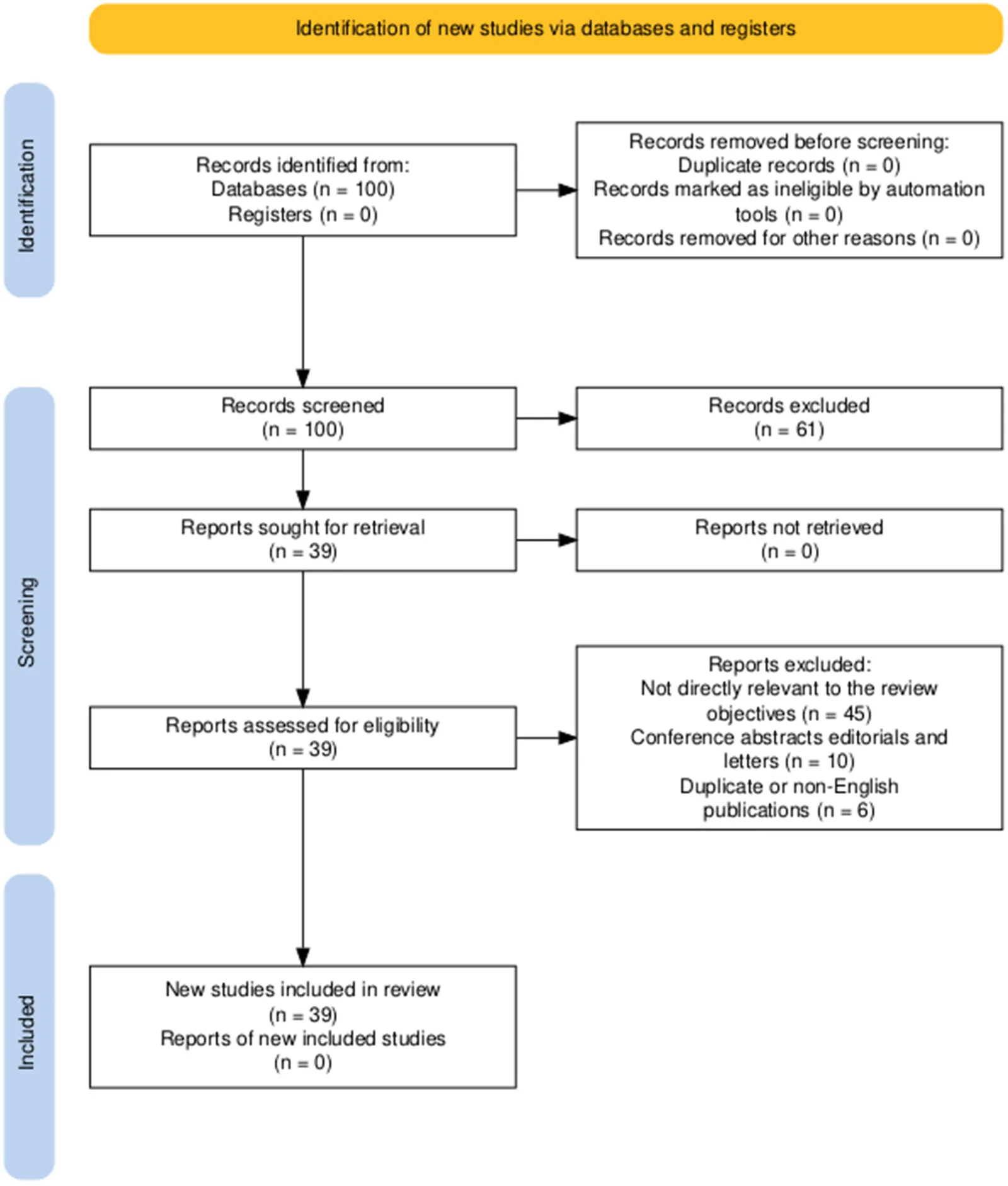
PRISMA-Style flow diagram of literature selection. The diagram summarizes the identification, screening, and selection of publications included in this narrative review. It is presented for transparency only and does not indicate full PRISMA compliance.

Approximately 100 records were initially identified. Following assessment for scientific relevance, clinical significance, and contribution to the study objectives, 39 references were selected for inclusion in the final evidence synthesis. Preference was given to peer-reviewed publications, particularly human interventional studies, randomized clinical trials, translational research, and contemporary clinical guidelines. Conference abstracts, duplicate publications, and studies not directly relevant to the review objectives were excluded. The literature-selection process is summarized in a PRISMA-style flow diagram adapted for the narrative-review design (Figure 2).

The selected literature was evaluated to address two principal research questions: (i) whether peptide therapeutics may represent viable alternatives to conventional strategies for restoring testicular function following testosterone replacement therapy–induced suppression, and (ii) whether peptide-based interventions may provide fertility-preserving therapeutic alternatives to conventional testosterone replacement therapy in men with hypogonadism.

Evidence was synthesized narratively and organized into thematic domains, including the pathophysiology of testosterone-induced reproductive suppression, established recovery strategies, emerging peptide therapeutics, recovery of endogenous HPT-axis function, and future directions for peptide-based interventions in male reproductive medicine.

## 3 Biological basis of TRT-induced reproductive suppression

Testosterone replacement therapy (TRT) remains the cornerstone treatment for male hypogonadism and effectively improves symptoms associated with testosterone deficiency, including reduced libido, erectile dysfunction, fatigue, depressed mood, decreased muscle mass, and impaired quality of life (1–4). Despite these benefits, exogenous testosterone disrupts the physiological regulation of the hypothalamic–pituitary–testicular (HPT) axis through negative feedback mechanisms (1, 9–11).

Under normal physiological conditions, gonadotropin-releasing hormone (GnRH) is secreted in a pulsatile manner by the hypothalamus, stimulating pituitary release of luteinizing hormone (LH) and follicle-stimulating hormone (FSH). LH promotes testosterone synthesis by Leydig cells, whereas FSH supports Sertoli cell function and spermatogenesis (25–27). This tightly regulated system is maintained through feedback actions of testosterone and estradiol on the hypothalamus and pituitary gland (26, 27).

Administration of exogenous testosterone suppresses endogenous GnRH, LH, and FSH secretion, resulting in reduced Leydig and Sertoli cell stimulation (9–12). Although serum testosterone concentrations may remain within or above the physiological range, intratesticular testosterone concentrations decline substantially. This distinction is clinically important because maintenance of spermatogenesis requires intratesticular testosterone levels that are considerably higher than circulating serum concentrations (12, 13, 15).

Consequently, prolonged gonadotropin suppression may impair spermatogenesis, reduce sperm production, decrease testicular volume, and compromise fertility (9–18). The degree of reproductive suppression varies among individuals and is influenced by factors such as age, treatment duration, baseline reproductive status, underlying etiology of hypogonadism, and concomitant medical conditions (9, 12, 17, 18). Recovery following TRT discontinuation is also influenced by baseline testicular reserve, semen parameters, and the duration of previous testosterone exposure. In general, younger patients, shorter TRT duration, preserved testicular volume, and normal baseline reproductive function are associated with a greater likelihood of recovery, whereas prolonged androgen exposure or pre-existing testicular dysfunction may delay or limit restoration of spermatogenesis (38). These factors should be considered when counselling patients and when selecting individualized fertility-restoration strategies. As a result, fertility preservation has become a major consideration in the management of men with functional hypogonadism and those desiring future fertility, particularly those of reproductive age (2, 4, 11, 21, 22).

Current management strategies for TRT-induced reproductive suppression include human chorionic gonadotropin (hCG), gonadotropins, selective estrogen receptor modulators (SERMs), and aromatase inhibitors (10, 11, 13–21). While these approaches may facilitate recovery of endogenous HPT-axis function in selected patients, treatment responses are variable and recovery may be incomplete or prolonged (10, 11, 14–20). Moreover, these interventions largely address downstream endocrine consequences rather than the upstream neuroendocrine regulators that govern reproductive function.

Advances in reproductive neuroendocrinology have identified several peptide-mediated pathways involved in regulation of the HPT axis, particularly kisspeptin, gonadotropin-releasing hormone (GnRH), neurokinin B, and KNDy neuron signaling (23–29). These pathways have emerged as potential therapeutic targets capable of modulating endogenous reproductive signaling and supporting HPT-axis function. Consequently, peptide therapeutics have attracted increasing interest as potential strategies for both management of TRT-induced reproductive suppression and fertility-preserving management of functional male hypogonadism (23, 24, 28, 30–35).

## 4 Limitations of conventional recovery strategies

Conventional therapies remain the current standard approach for fertility preservation and restoration in men with TRT-induced reproductive suppression. Human chorionic gonadotropin (hCG), follicle-stimulating hormone (FSH), and selective estrogen receptor modulators (SERMs) have demonstrated clinical efficacy in appropriately selected patients and continue to represent first-line therapeutic options. Reported success rates vary considerably according to patient characteristics, treatment protocols, and the underlying cause of hypogonadism, emphasizing the importance of individualized management. The increasing recognition of TRT-induced reproductive suppression has led to the use of several strategies aimed at preserving fertility and supporting recovery of endogenous HPT-axis function. Current approaches include human chorionic gonadotropin (hCG), gonadotropins such as follicle-stimulating hormone (FSH) and human menopausal gonadotropin (hMG), selective estrogen receptor modulators (SERMs), and, in selected cases, aromatase inhibitors (10, 11, 13–21). Although these interventions have clinical utility, their effectiveness is variable and their long-term application remains limited by heterogeneous protocols, patient-specific responses, cost, tolerability, and incomplete recovery of spermatogenesis (10, 11, 14–20).

hCG is among the most commonly used interventions for prevention or treatment of TRT-induced testicular suppression. By mimicking luteinizing hormone activity, hCG stimulates Leydig cells and helps maintain intratesticular testosterone production (13, 15, 16). However, hCG therapy does not consistently restore spermatogenesis in all patients and may be limited by injection burden, increased estradiol concentrations, gynecomastia, variable dosing regimens, and treatment cost (14–16).

Gonadotropin-based therapies, including FSH and hMG, are frequently used in fertility-focused protocols, particularly in men with more severe impairment of spermatogenesis (14, 16, 21). While these agents can improve reproductive outcomes in selected patients, their use is constrained by prolonged treatment duration, expense, treatment complexity, and inconsistent clinical responses (14, 16, 21).

SERMs, including clomiphene citrate and related agents, stimulate endogenous gonadotropin secretion by modulating estrogen-mediated negative feedback at the hypothalamic–pituitary level (19, 20). These agents are attractive because they may increase endogenous testosterone production while avoiding direct suppression of spermatogenesis. Nevertheless, outcomes remain variable, and concerns persist regarding long-term efficacy, adverse effects, patient tolerability, and durability of response after treatment discontinuation (18–20). Furthermore, clinical responses vary according to the underlying etiology of hypogonadism and the degree of HPT-axis suppression (4, 5, 18).

Aromatase inhibitors may be considered in selected men, particularly those with elevated estradiol levels or obesity-associated hormonal imbalance. However, their role in recovery of TRT-induced reproductive suppression is less clearly established, and the supporting evidence remains more limited than for hCG, gonadotropins, or SERMs (18, 20).

Collectively, conventional recovery strategies remain important in clinical practice but are limited by inconsistent clinical efficacy and by their focus on downstream endocrine manipulation rather than modulation of upstream neuroendocrine regulation (10, 11, 14–21). This limitation has stimulated interest in peptide therapeutics that target central regulators of the hypothalamic–pituitary–testicular axis, including kisspeptin, GnRH, neurokinin B, and KNDy neuron signaling (23–29). By targeting endogenous reproductive pathways, these agents may offer a more physiological strategy for supporting endogenous HPT-axis function and may eventually provide fertility-preserving alternatives or adjuncts to conventional testosterone replacement therapy (23, 24, 28, 30–35).

A comparative overview of conventional fertility-preserving strategies and emerging peptide-based therapeutic approaches is presented in Table 1.

**Table 1:** Comparison of conventional fertility-preserving strategies and emerging peptide-based therapeutic approaches.

Therapy	Primary Target	Main Advantages	Main Limitations	Evidence for Fertility Restoration	Current Clinical Status
hCG	LH receptor (Leydig cells)	Maintains intratesticular testosterone; widely used	Injections, cost, gynecomastia, variable recovery	Moderate clinical evidence	Established clinical practice (13–16)
FSH/hMG	Sertoli cells/spermatogenesis	May improve spermatogenesis in selected patients	Expensive, prolonged treatment, variable response	Moderate clinical evidence	Established clinical practice (14, 16, 21)
SERMs	Estrogen receptor (hypothalamus/pituitary)	Stimulates endogenous LH/FSH; oral administration	Variable response; limited long-term data	Moderate clinical evidence	Established clinical practice (18–20)
Aromatase inhibitors	Aromatase enzyme	Useful in selected men with elevated estradiol	Limited evidence; selected indications only	Limited clinical evidence	Selected patients (18, 20)
Kisspeptin	KISS1R → GnRH neurons	Physiological activation of the HPT axis; preserves endogenous signaling	No TRT-specific trials; early-phase studies	Indirect human physiological evidence; no TRT-specific fertility trials	Investigational (23, 24, 28, 30–35)
GnRH/Gonadorelin	GnRH receptor/pituitary	Physiological stimulation of LH/FSH	Pulsatile administration required; limited practicality	Limited clinical evidence	Limited clinical use (25–27, 34)
Neurokinin B/KNDy peptides	Upstream GnRH regulation	Novel neuroendocrine targets	Predominantly preclinical or early translational evidence	No direct fertility evidence	Experimental (23–25, 27)

## 5 Emerging peptide therapeutics and clinical evidence

### 5.1 Kisspeptin and kisspeptin analogues

Kisspeptin has emerged as one of the most promising peptide-based therapeutic targets in reproductive medicine because of its central role in regulating gonadotropin-releasing hormone (GnRH) secretion and activation of the hypothalamic–pituitary–testicular (HPT) axis (23–26). Through binding to the KISS1 receptor (KISS1R/GPR54), kisspeptin stimulates hypothalamic GnRH release, leading to downstream secretion of luteinizing hormone (LH) and follicle-stimulating hormone (FSH), ultimately promoting testicular steroidogenesis and spermatogenesis (23–26, 28, 29).

The physiological role of kisspeptin in regulating the hypothalamic–pituitary–testicular axis has been summarized in the Introduction; therefore, this section focuses on the available experimental and clinical evidence supporting its therapeutic potential in TRT-induced reproductive suppression.

Human studies have provided important proof-of-concept evidence supporting the therapeutic potential of kisspeptin. Dhillo et al. demonstrated that kisspeptin-54 administration significantly stimulated the HPT axis in healthy men, resulting in increased gonadotropin secretion (28). These findings provide physiological proof of concept but do not constitute direct evidence of restoration of spermatogenesis or fertility following TRT-induced suppression. Subsequently, George et al. reported that kisspeptin-10 increased LH pulse frequency and LH secretion in healthy men, supporting its role in physiological GnRH pulse generation (30). Additional studies demonstrated preserved hypothalamic and pituitary responsiveness to kisspeptin in healthy older men, suggesting that age-related changes do not necessarily impair kisspeptin-mediated reproductive signaling (31).

Clinical observations have also extended beyond healthy volunteers. In men with type 2 diabetes and mild biochemical hypogonadism, kisspeptin-10 significantly increased LH and testosterone concentrations, highlighting its potential therapeutic relevance in hypogonadal states (33). Furthermore, direct comparison of kisspeptin-10, kisspeptin-54, and GnRH demonstrated robust stimulation of gonadotropin secretion by both kisspeptin isoforms, further supporting their capacity to activate endogenous reproductive pathways (34).

More recently, a randomized clinical trial demonstrated that kisspeptin administration enhanced sexual brain processing and penile tumescence in men with hypoactive sexual desire disorder (32). Although these findings support the broader neuroendocrine effects of kisspeptin, they do not provide direct evidence for restoration of spermatogenesis or fertility following TRT-induced reproductive suppression. Additional studies have confirmed stimulation of reproductive hormone secretion without significant adverse neuropsychiatric effects, further supporting the safety profile of kisspeptin-based interventions (35). A summary of the principal human interventional studies evaluating kisspeptin in men is presented in Table 2.

**Table 2:** Human interventional studies evaluating kisspeptin in men.

Ref. No.	Study	Design	Population	Intervention	Main findings
(28)	Dhillo et al., 2005	Interventional physiological study	Healthy men	Kisspeptin-54 administration	Significant stimulation of the hypothalamic–pituitary–gonadal (HPG) axis with increased LH secretion.
(30)	George et al., 2011	Interventional physiological study	Healthy men	Kisspeptin-10 administration	Increased LH pulse frequency and LH secretion, supporting a role in GnRH pulse generation.
(31)	Abbara et al., 2018	Interventional study	Healthy older men	Kisspeptin-54 and GnRH administration	Preserved hypothalamic and pituitary responsiveness to kisspeptin and GnRH despite aging.
(33)	George et al., 2013	Interventional study	Men with type 2 diabetes and mild hypogonadism	Kisspeptin-10 administration	Increased LH and testosterone concentrations, suggesting therapeutic potential in hypogonadal states.
(34)	Jayasena et al., 2015	Comparative interventional study	Healthy men	Intravenous kisspeptin-10, kisspeptin-54, and GnRH	Demonstrated gonadotropin stimulation by both kisspeptin isoforms and enabled comparison with GnRH.
(35)	Mills et al., 2025	Interventional study	Healthy adults	Kisspeptin administration	Increased reproductive hormone secretion without significant anxiety-related adverse effects.
(32)	Mills et al., 2023	Randomized Clinical Trial (RCT)	Men with hypoactive sexual desire disorder	Kisspeptin-54 vs. placebo	Enhanced sexual brain processing and penile tumescence, supporting translational therapeutic potential.

To improve clinical applicability, several kisspeptin analogues with enhanced pharmacokinetic properties and prolonged biological activity are currently under investigation (23, 24). Although long-term clinical evidence remains limited, kisspeptin-based therapies represent one of the most extensively investigated peptide approaches currently under evaluation for restoration of endogenous reproductive function and fertility-preserving management of male hypogonadism.

Despite its strong physiological rationale, current evidence supporting kisspeptin for TRT-induced reproductive suppression remains limited. Most available human studies have evaluated acute endocrine responses rather than fertility restoration, and no large randomized clinical trials have specifically investigated kisspeptin in men with TRT-induced infertility. Therefore, its clinical application should currently be considered investigational until larger studies establish its long-term efficacy, safety, optimal dosing, and effects on spermatogenesis and fertility outcomes.

### 5.2 Gonadotropin-releasing hormone (GnRH) and gonadorelin-based therapeutics

GnRH represents one of the earliest peptide-based therapeutic approaches for restoration of reproductive function. Under physiological conditions, pulsatile GnRH secretion stimulates pituitary release of LH and FSH, thereby maintaining testicular steroidogenesis and spermatogenesis (25–27). Therapeutic administration of GnRH or its analogue gonadorelin can restore gonadotropin secretion through activation of endogenous reproductive signaling pathways rather than direct hormonal replacement.

From a mechanistic perspective, GnRH-based therapy provides important proof-of-concept evidence that reproductive function can be restored through upstream neuroendocrine stimulation. Comparative studies have demonstrated that kisspeptin-induced gonadotropin secretion ultimately converges upon GnRH-dependent signaling pathways, highlighting the central role of GnRH within the HPT axis (34). However, despite its physiological rationale, widespread clinical use of GnRH-based therapy remains limited by practical challenges related to pulsatile administration requirements, treatment complexity, and long-term feasibility. The therapeutic efficacy of GnRH depends on physiological pulsatile administration, whereas continuous GnRH exposure results in receptor downregulation and suppression of gonadotropin secretion.

Although pulsatile GnRH therapy has demonstrated efficacy in selected patients with hypothalamic hypogonadism, its clinical application remains limited by the need for specialized infusion pumps, high cost, limited availability, and the requirement for careful patient selection and monitoring. Consequently, GnRH therapy is currently reserved for specific clinical indications rather than routine management of TRT-induced reproductive suppression.

### 5.3 Neurokinin B, KNDy signaling, and future peptide targets

Recent advances in reproductive neuroendocrinology have identified neurokinin B and KNDy (kisspeptin-neurokinin B-dynorphin) neurons as critical regulators of pulsatile GnRH secretion (23–25, 27). Located primarily within the hypothalamic arcuate nucleus, KNDy neurons coordinate reproductive hormone pulsatility through complex interactions involving kisspeptin, neurokinin B, and dynorphin signaling (25, 27).

Recognition of the KNDy network has expanded understanding of reproductive regulation and identified several novel therapeutic targets upstream of GnRH secretion (23–25). Neurokinin B signaling appears particularly important in regulating GnRH pulse generation, and modulation of this pathway may eventually provide additional opportunities for selective control of reproductive endocrine function (24, 27). Although clinical evidence remains limited, these pathways represent promising targets for future fertility-preserving interventions and may complement existing peptide-based therapeutic strategies.

### 5.4 Other emerging peptide candidates

Beyond kisspeptin, GnRH-based therapies, and neurokinin signaling modulators, additional peptide candidates are being explored in reproductive medicine. Recent attention has focused on metabolic peptides, particularly glucagon-like peptide-1 (GLP-1) receptor agonists, because of their potential interactions with metabolic and reproductive pathways (36, 37). Although current evidence primarily relates to indirect effects mediated through weight reduction, metabolic improvement, and hormonal regulation, these agents may influence male reproductive health in selected populations (36, 37).

Advances in peptide engineering and molecular design are expected to accelerate development of next-generation reproductive peptides with improved selectivity, stability, and therapeutic efficacy (23, 24). While most remain investigational, these emerging compounds may broaden future treatment options aimed at restoration of endogenous reproductive function and fertility preservation.

## 6 Discussion

### 6.1 From hormone replacement to biological restoration

The findings reviewed in this article suggest that peptide therapeutics may represent a promising therapeutic approach in male reproductive medicine. Whereas conventional testosterone replacement therapy effectively alleviates symptoms of hypogonadism, it frequently suppresses endogenous reproductive function through negative feedback on the hypothalamic–pituitary–testicular axis (1, 2, 9–18). In contrast, peptide-based therapies aim to support physiological reproductive signaling by targeting upstream neuroendocrine regulators, thereby promoting endogenous testosterone production while potentially preserving fertility (23–35).

### 6.2 Why kisspeptin is the most promising candidate

Among currently available peptide candidates, kisspeptin currently has the most extensive human clinical evidence among peptide-based therapeutic candidates. Human interventional studies have consistently demonstrated stimulation of LH, FSH, and testosterone secretion following kisspeptin administration (28, 30–35). Furthermore, the randomized clinical trial by Mills et al. demonstrated favorable effects on sexual brain processing and penile tumescence in men with hypoactive sexual desire disorder (32). However, this study did not evaluate fertility restoration or recovery from TRT-induced reproductive suppression and should therefore be interpreted as supportive neuroendocrine evidence rather than direct evidence for fertility-preserving efficacy.

### 6.3 Potential role in TRT-suppressed testicular function

Although no large clinical trials have specifically evaluated kisspeptin for recovery of TRT-suppressed testicular function, the available evidence suggests that activation of endogenous GnRH-LH signaling may represent a biologically plausible strategy for supporting recovery of endogenous HPT-axis function. Unlike hCG and gonadotropin-based approaches that primarily replace downstream hormonal signals, kisspeptin acts at the apex of reproductive neuroendocrine regulation and may therefore offer a more physiological mechanism for reactivation of the HPT axis (23–35). Peptide-based interventions should be considered separately during ongoing TRT and after TRT discontinuation, as spontaneous recovery may occur following treatment withdrawal (9, 12).

### 6.4 Potential role in male hypogonadism

Beyond reproductive recovery, peptide therapeutics may eventually provide fertility-preserving therapeutic alternatives for selected men with functional male hypogonadism. Current TRT guidelines acknowledge the challenge of balancing symptom improvement with preservation of fertility (1, 2, 4, 21). Because kisspeptin stimulates endogenous testosterone production without directly replacing testosterone, it may offer advantages in younger men who desire future fertility (28, 30–35).

The therapeutic potential of peptide-based interventions is likely to vary according to the underlying cause of hypogonadism. Future studies should evaluate these therapies across specific hypogonadal subgroups rather than considering male hypogonadism as a single homogeneous condition (4, 5).

### 6.5 Current limitations of the evidence

Although multiple human interventional studies demonstrate stimulation of LH and testosterone secretion by kisspeptin, evidence remains limited to early-phase clinical investigations, and large randomized controlled trials evaluating long-term efficacy and fertility outcomes in hypogonadal men are still lacking (28, 30–35).

Additional limitations include small sample sizes, heterogeneous study populations, variable dosing regimens, and limited long-term follow-up.

### 6.6 Future directions

Future research should focus on randomized controlled trials evaluating long-term reproductive outcomes, fertility preservation, symptom improvement, quality of life, and direct comparisons with established recovery strategies such as hCG, SERMs, and gonadotropins (10, 11, 13–21). Development of long-acting kisspeptin analogues may further enhance clinical applicability and facilitate translation into routine practice (23, 24). Future studies should also establish optimal dosing strategies, evaluate long-term safety, and determine whether receptor desensitization or tachyphylaxis may occur during prolonged peptide administration (23, 24).

Although the present review focuses primarily on neuroendocrine peptide therapeutics acting through the HPT axis, future research may also explore testis-targeted peptides capable of directly modulating Leydig cell, Sertoli cell, or germ-cell function independent of central neuroendocrine regulation (13, 25, 27).

## 7 Conclusions

Peptide therapeutics represent a promising emerging strategy in male reproductive medicine by targeting the neuroendocrine mechanisms that regulate reproductive function. Unlike conventional testosterone replacement therapy, these agents may support endogenous hypothalamic–pituitary–testicular axis activity while preserving physiological reproductive signaling.

Among currently investigated candidates, kisspeptin demonstrates the strongest translational and clinical evidence, with human studies consistently showing activation of gonadotropin secretion and endogenous testosterone production. Additional targets, including gonadotropin-releasing hormone, neurokinin B, and KNDy neuron signaling pathways, further support the concept of neuroendocrine modulation as a potential therapeutic approach.

Although current evidence remains limited to early-stage clinical investigations, peptide-based therapies offer a biologically plausible strategy for supporting recovery of endogenous HPT-axis function following TRT-induced reproductive suppression and for fertility-preserving management of selected men with functional male hypogonadism. Future randomized clinical trials are required to establish long-term efficacy, safety, reproductive outcomes, and clinical applicability.

Overall, peptide therapeutics may contribute to future therapeutic strategies aimed at supporting endogenous reproductive function alongside existing treatments, representing a promising direction for future research in the management of male hypogonadism and reproductive health.

## Key message

Peptides represent a promising fertility-preserving strategy for restoration of endogenous reproductive function in men with hypogonadism and TRT-induced reproductive suppression.
