# Supplementary material for: Emerging peptide and neuroendocrine strategies for TRT-induced reproductive suppression and functional male hypogonadism: a narrative review
Source: Front Reprod Health. 2026 Aug 14;8:1914709. doi: 10.3389/frph.2026.1914709 (PMC13522740; doi:10.3389/frph.2026.1914709)
Supplement: Supplementary file 1 [file Supplementaryfile1.pdf]

Supplementary Material

1 Supplementary Figures and Tables

1.1 Supplementary Figures

1.1.1 Supplementary Figure 1.

Figure 1. The Emerging Therapeutic Transition in Male Reproductive Medicine: From Hormone Replacement to Biological Restoration. *Original figure created by the second author AAMK*

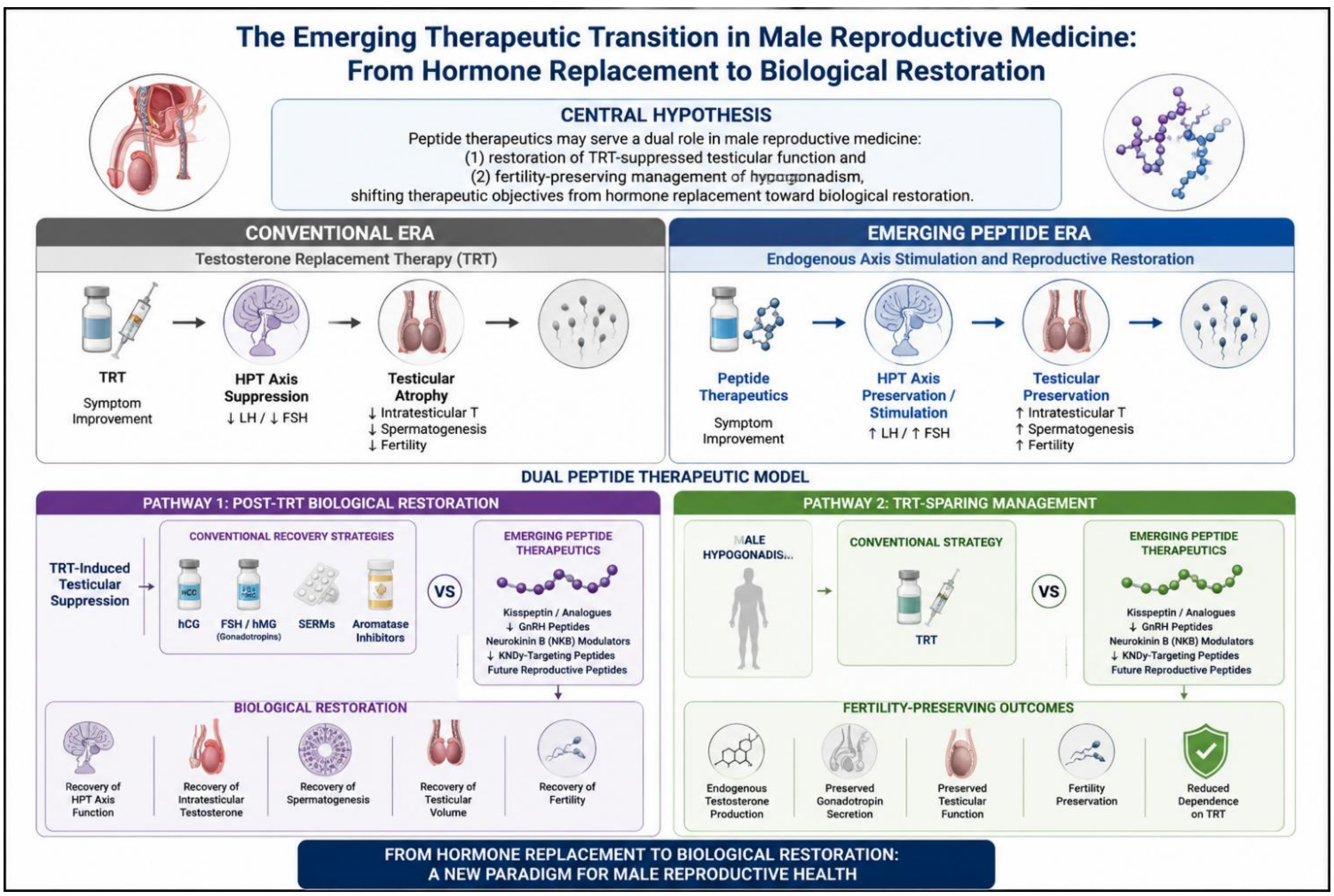

1.1.2 Supplementary Figure 2.

Figure 2. PRISMA-Style Flow Diagram of Literature Selection. The diagram summarizes the identification, screening, and selection of publications included in this narrative review. It is presented for transparency only and does not indicate full PRISMA compliance.

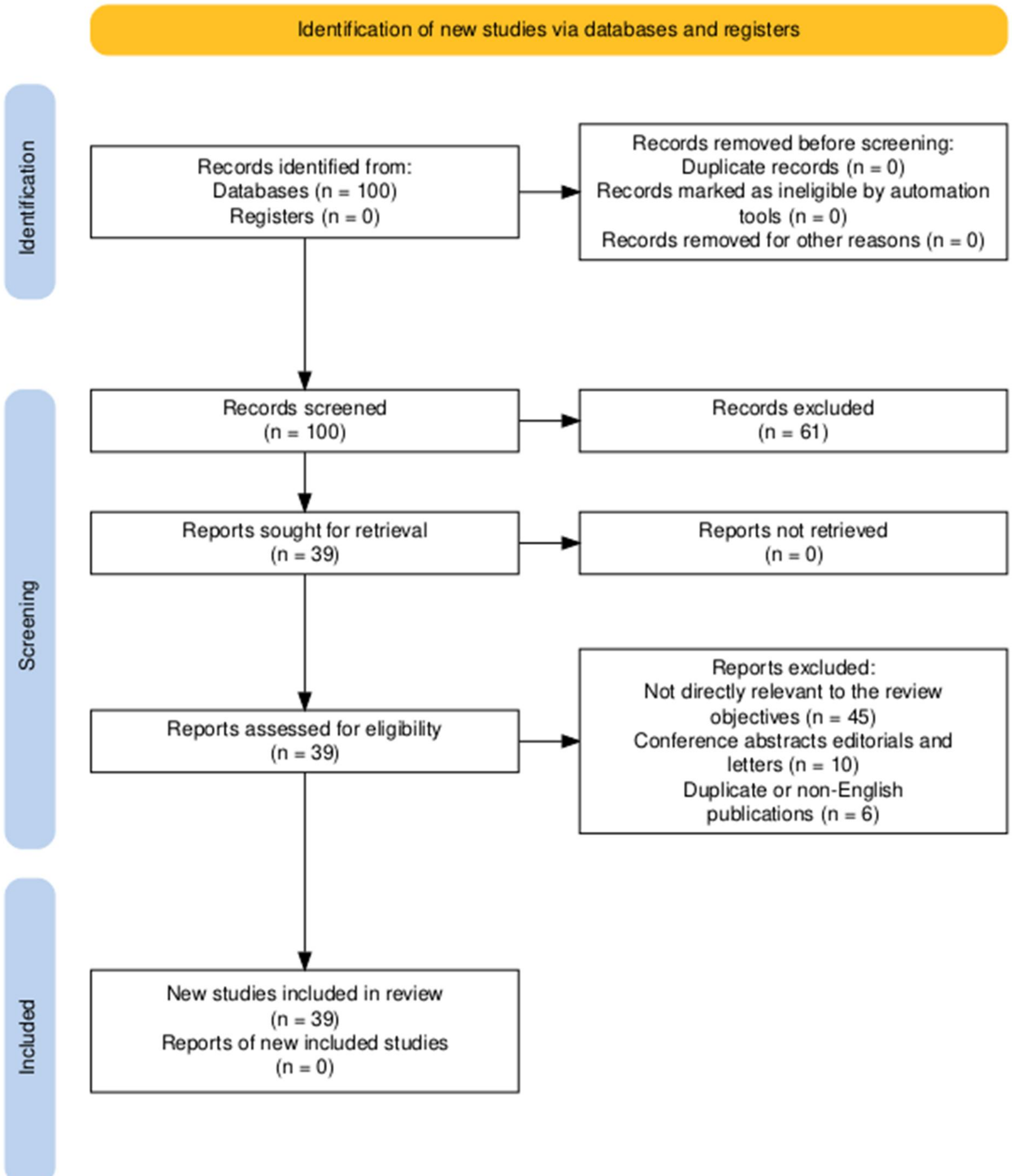

1.2 Supplementary Tables

1.2.1 Supplementary Table 1. Comparison of Conventional Fertility-Preserving Strategies and Emerging Peptide-Based Therapeutic Approaches. *Original table created by the second author AAMK.*

Therapy	Primary Target	Main Advantages	Main Limitations	Evidence for Fertility Restoration	Current Clinical Status
hCG	LH receptor (Leydig cells)	Maintains intratesticular testosterone; widely used	Injections, cost, gynecomastia, variable recovery	Moderate clinical evidence	Established clinical practice [11–14]
FSH / hMG	Sertoli cells / spermatogenesis	May improve spermatogenesis in selected patients	Expensive, prolonged treatment, variable response	Moderate clinical evidence	Established clinical practice [12,14,21]
SERMs	Estrogen receptor (hypothalamus/pituitary)	Stimulates endogenous LH/FSH; oral administration	Variable response; limited long-term data	Moderate clinical evidence	Established clinical practice [16,19,20]
Aromatase inhibitors	Aromatase enzyme	Useful in selected men with elevated estradiol	Limited evidence; selected indications only	Limited clinical evidence	Selected patients [16,20]
Kisspeptin	KISS1R → GnRH neurons	Physiological activation of the HPT axis; preserves endogenous signaling	No TRT-specific trials; early-phase studies	Indirect human physiological evidence; no TRT-specific fertility trials	Investigational [1,2,5,32–37]
GnRH / Gonadorelin	GnRH receptor / pituitary	Physiological stimulation of LH/FSH	Pulsatile administration required; limited practicality	Limited clinical evidence	Limited clinical use [3,4,31,36]
Neurokinin B / KNDy peptides	Upstream GnRH regulation	Novel neuroendocrine targets	Predominantly preclinical or early translational evidence	No direct fertility evidence	Experimental [1–3,31]

1.2.2 **Supplementary Table 2.** Human Interventional Studies Evaluating Kisspeptin in Men.

Original table created by the second author AAMK.

Ref. No.	Study	Design	Population	Intervention	Main Findings

5	Dhillo et al., 2005	Interventional physiological study	Healthy men	Kisspeptin-54 administration	Significant stimulation of the hypothalamic–pituitary–gonadal (HPG) axis with increased LH secretion.
32	George et al., 2011	Interventional physiological study	Healthy men	Kisspeptin-10 administration	Increased LH pulse frequency and LH secretion, supporting a role in GnRH pulse generation.
33	Abbara et al., 2018	Interventional study	Healthy older men	Kisspeptin-54 and GnRH administration	Preserved hypothalamic and pituitary responsiveness to kisspeptin and GnRH despite aging.
35	George et al., 2013	Interventional study	Men with type 2 diabetes and mild hypogonadism	Kisspeptin-10 administration	Increased LH and testosterone concentrations, suggesting therapeutic potential in hypogonadal states.
36	Jayasena et al., 2015	Comparative interventional study	Healthy men	Intravenous kisspeptin-10, kisspeptin-54, and GnRH	Demonstrated gonadotropin stimulation by both kisspeptin isoforms and enabled comparison with GnRH.
37	Mills et al., 2025	Interventional study	Healthy adults	Kisspeptin administration	Increased reproductive hormone secretion without significant effects on anxiety.
34	Mills et al., 2023	Randomized Clinical Trial (RCT)	Men with hypoactive sexual desire disorder	Kisspeptin-54 vs placebo	Enhanced sexual brain processing and penile tumescence, supporting translational therapeutic potential.
